# Application of Laser Treatment in MOS-TFT Active Layer Prepared by Solution Method

**DOI:** 10.3390/mi12121496

**Published:** 2021-11-30

**Authors:** Nanhong Chen, Honglong Ning, Zhihao Liang, Xianzhe Liu, Xiaofeng Wang, Rihui Yao, Jinyao Zhong, Xiao Fu, Tian Qiu, Junbiao Peng

**Affiliations:** 1 Institute of Polymer Optoelectronic Materials and Devices, State Key Laboratory of Luminescent Materials and Devices, South China University of Technology, Guangzhou 510640, China; chen-nanhong@foxmail.com (N.C.); ninghl@scut.edu.cn (H.N.); 201530291443@mail.scut.edu.cn (Z.L.); 202010103138@mail.scut.edu.cn (J.Z.); 201630343721@mail.scut.edu.cn (X.F.); psjbpeng@scut.edu.cn (J.P.); 2 Research Center of Flexible Sensing Materials and Devices, School of Applied Physics and Materials, Wuyi University, Jiangmen 529020, China; msliuxianzhe@mail.scut.edu.cn; 3 Institute of Semiconductors, Chinese Academy of Sciences, Beijing 100083, China; wangxiaofeng@semi.ac.cn; 4 Department of Intelligent Manufacturing, Wuyi University, Jiangmen 529020, China

**Keywords:** solution method, laser treatment, active layer, metal oxide semiconductor thin film transistor

## Abstract

The active layer of metal oxide semiconductor thin film transistor (MOS-TFT) prepared by solution method, with the advantages of being a low cost and simple preparation process, usually needs heat treatment to improve its performance. Laser treatment has the advantages of high energy, fast speed, less damage to the substrate and controllable treatment area, which is more suitable for flexible and large-scale roll-to-roll preparation than thermal treatment. This paper mainly introduces the basic principle of active layer thin films prepared by laser treatment solution, including laser photochemical cracking of metastable bonds, laser thermal effect, photoactivation effect and laser sintering of nanoparticles. In addition, the application of laser treatment in the regulation of MOS-TFT performance is also described, including the effects of laser energy density, treatment atmosphere, laser wavelength and other factors on the performance of active layer thin films and MOS-TFT devices. Finally, the problems and future development trends of laser treatment technology in the application of metal oxide semiconductor thin films prepared by solution method and MOS-TFT are summarized.

## 1. Introduction

At present, new display technology products are endlessly emerging. People continue to have higher requirements for the characteristics of display devices, such as high resolution, thin, flexible, transparent, rich color and so on. Metal oxide semiconductor thin film transistor (MOS-TFT) has the advantages of high mobility (1–100 cm^2^/Vs) and good film uniformity [1,2,3,4,5]. It has become a strong competitor in the display backplane industry represented by active matrix liquid crystal display and active matrix organic light emitting diode.

**Figure 1:**
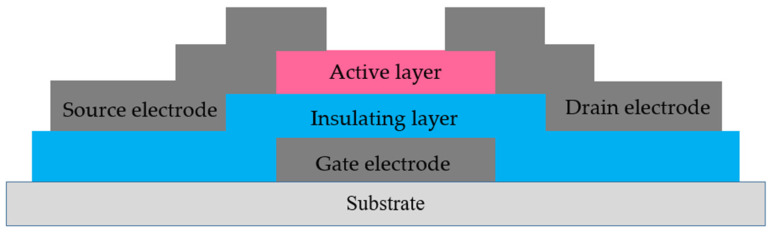
Schematic diagram of TFT device structure.

Thin film transistor is a kind of field effect transistor. TFT devices are usually composed of active layers, insulating layers, gate electrodes, source electrodes and drain electrodes, the common TFT device structure is shown in Figure 1. In TFT, the material that plays the most important role is the semiconductor active layer. According to the difference of semiconductor active layer materials, TFT can be divided into the following four categories: a-Si TFT, p-Si TFT, OTFT and MOS-TFT [6,7,8,9]. Among them, MOS-TFT has the advantages of high field effect mobility, high uniformity, good electrical stability and high transparency, which is suitable for the future display preparation requirements, such as large size and flexibility [1,4,10].

**Figure 2:**
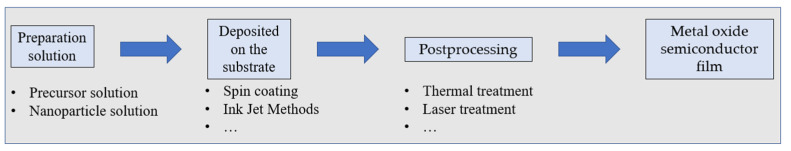
Schematic diagram indicating a typical solution process synthesis of metal oxide semiconductor thin films and the corresponding TFT devices.

Solution-processed deposition offers the advantages of a simple process, high-throughput, high material utilization rate, and easy control of chemical components, which provides the possibility for large-area preparation of metal oxide semiconductor [10,11,12,13,14]. In the study of solution preparation of MOS-TFT, the active layer is mainly made of precursor prepared by sol–gel [15,16] or nanoparticles (NPs) dispersed in carrier solvent [17,18,19], which are deposited on the substrate by spin coating method, inkjet printing method and so on. Whether the thin films are prepared by sol–gel method or nano-particle method, the precursors or nanostructures usually need postprocessing to improve their properties [20]. The typical process of preparing metal oxide semiconductor thin films and corresponding TFT devices by solution method is shown in Figure 2.

The traditional thermal treatment process has some disadvantages, such as high energy consumption, long treatment time, high process temperature and incompatibility of flexible substrates [21]. In order to solve this problem, various studies have tried to reduce the treatment temperature by compensating for other energy sources (for example, optical, chemical and physical methods), rather than reducing the activation energy. Many researchers reduce the treatment temperature of the active layer and MOS-TFT prepared by solution method by microwave treatment [22,23,24], plasma treatment [23,25], ozone ultraviolet treatment (UV) [26,27,28,29,30], high pressure treatment [22,23,31,32], water based/hydrolysis [28,33], low temperature steam treatment [34] and so on. The common process parameters of low temperature treatment are shown in Table 1. However, these methods are not suitable for large-scale roll-to-roll (R2R) processes. On the R2R production line, the treatment time is limited by the length of the on-line curing furnace. For example, for the speed of 1 m min^−1^ and the oven length of 5 m, the curing time of each treatment layer is limited to 5 min [27,35,36,37], while these methods require a longer treatment time.

**Figure 3:**
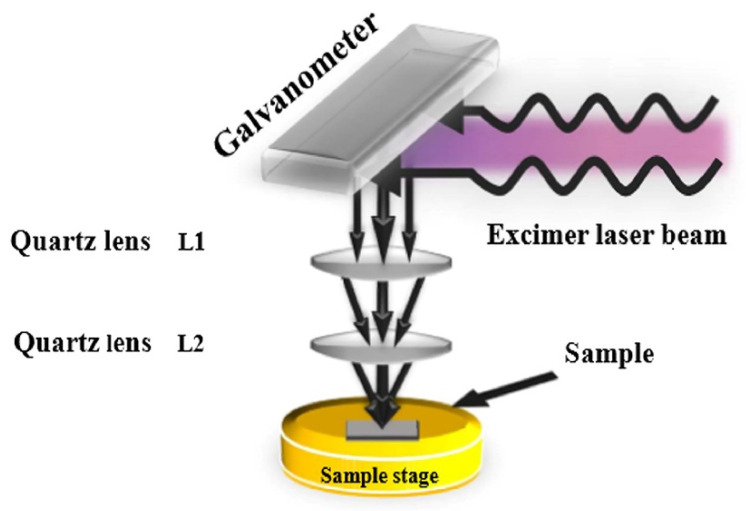
Schematic diagram of laser treatment device [48].

As a new treatment technology in the field of flexible, printing and wearable devices, laser treatment effectively avoids the shortcomings of other treatment methods, such as high energy consumption, long processing time, high process temperature, incompatibility with flexible substrate, only the whole device being treatable without the active layer being treated accurately. Laser treatment can effectively treat precursor films or nanoparticles through high-energy radiation and absorption of high-energy photons. By adjusting the laser processing parameters, such as laser intensity, pulse width and scanning speed, the energy input into the film can be accurately controlled to achieve the desired thermal effect [38,39,40,41,42,43]. In addition, the heating and cooling rate of laser treatment (>10^6^ °C s^−1^) is several orders of magnitude higher than that of conventional heat treatment and rapid thermal treatment, so that the thin films can be processed quickly with minimal energy loss [44]. Laser treatment is a top-down treatment technology and the treatment position can be accurately controlled, so the treatment area can be limited to a specific range of in-plane and thickness direction, and the thin films and nanostructures can be selectively treated to improve the properties of thin films and MOS-TFT without affecting the substrate and adjacent materials [43,45,46,47]. Common laser treatment equipment is shown in Figure 3 [48].

Laser treatment technology has many advantages and has made remarkable achievements in the application of active layer thin films and MOS-TFT devices prepared by solution method. However, there remains some shortcomings in the related research, such as less application on flexible substrates, less research on the influence of laser frequency and pulse number, and so on, which need to be further studied and improved.

## 2. Mechanism of Laser Treatment

Laser is a kind of high-energy beam with monochromaticity, coherence and collimation produced by stimulated emission process. In the process of laser treatment, the thin films are treated effectively through the thermal effect and photochemical reaction caused by the absorption of high-energy photons.

Lasers can be divided into solid-state lasers and excimer lasers according to working substances. The output beam of solid-state laser is usually Gaussian beam, and its energy curve is similar to Gaussian function curve. In contrast to solid-state lasers, the output beam of excimer lasers is usually flat-topped beam, and its energy density distribution is almost the same in a certain region.

According to the pulse width, laser can usually be divided into nanosecond laser, femtosecond laser and picosecond laser. Compared with nanosecond laser, femtosecond laser and picosecond laser can provide ultrashort pulse and low-energy high transient intensity, avoid damage to surrounding materials, and minimize thermal diffusion zone and light diffraction in ablated materials in high-resolution pattern making [49,50,51].

The laser wavelength and the band gap width of the material together determine the laser absorption mechanism of the material. The shorter the laser wavelength, the higher the laser photon energy. When the laser photon energy is higher than the material band gap, single photon absorption is the main mechanism of exciting valence electrons to the conduction band [51]. When the photon energy is lower than the material band gap or the single-photon absorption is suppressed by band filling, it is mainly multiphoton absorption [51].

### 2.1. Active Layer Thin Films Prepared by Sol-Gel Method

Unlike metal oxide films and MOS-TFT prepared by vacuum method (such as magnetron sputtering), there are impurities such as dissolved metal ligands (e.g., alkoxides, nitrates, chlorides), condensation by-products (e.g., water, alcohol), solvents and stabilizers in the precursor films prepared by solution method [29]. These impurities act as traps and hinder the effective formation of metal oxide framework, which plays the role of carrier channel. Therefore, in order to prepare high quality thin films, the removal of impurities is crucial. In order to prepare high quality metal oxide thin films, it is necessary not only to remove the impurities contained in the precursors, but also to provide enough energy to promote the Polycondensation and the densification of the thin films to form the metal-oxygen-metal (M-O-M) lattice structure [52,53,54,55]. The improvement of the lattice structure and the densification of the thin film help to reduce the traps and the potential barrier, increase the carrier mobility, and then improve the device performance of MOS-TFT [43,47,53,54,56,57,58,59,60].

**Figure 4:**
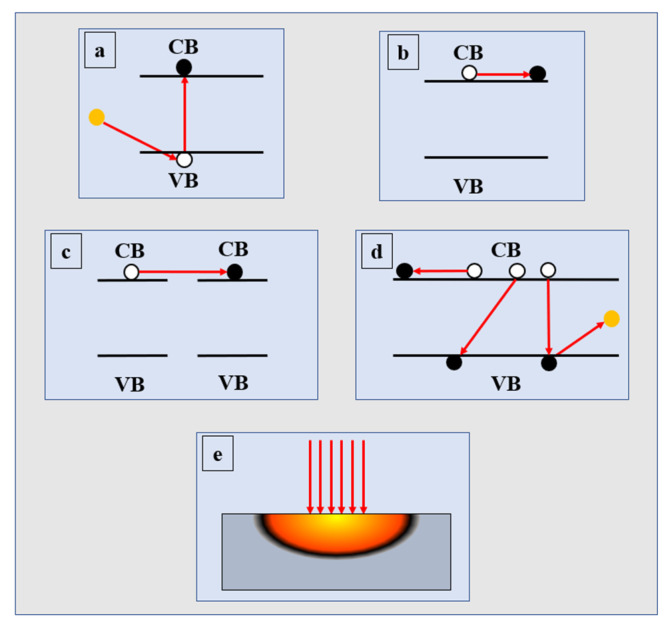
Schematic diagram of laser thermal effect: (**a**) photon absorption and carrier excitation; (**b**) carrier–carrier scattering; (**c**) carrier-phonon scattering; (**d**) carrier recombination; (**e**) thermal effect and thermal diffusion.

**Figure 5:**
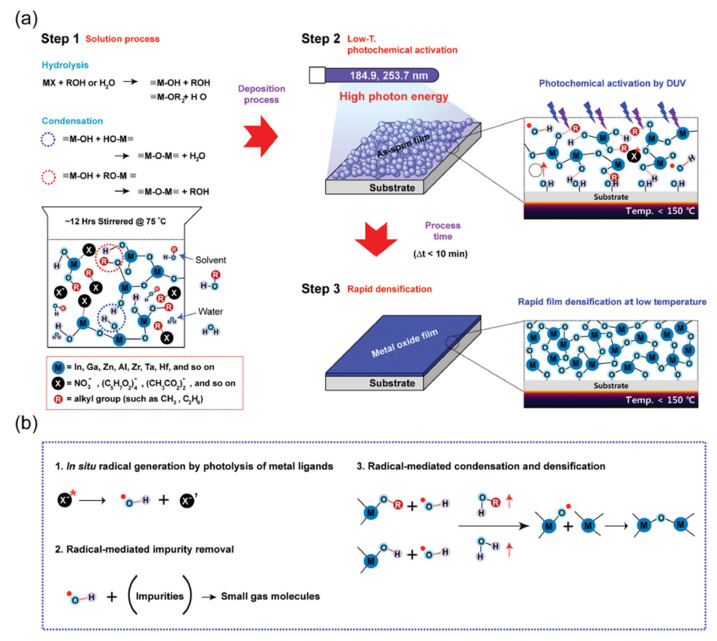
Photoactivation of sol–gel metal oxide materials and the proposed mechanism: (**a**) overall schematic illustration of the rapid low-temperature photoactivation of various sol–gel metal oxide films; (**b**) proposed physicochemical mechanism of the rapid low-temperature photoactivation process via photochemical activation (direct photodecomposition of impurities, in situ radical formation, enhancement of rapid condensation and densification) [29].

Laser treatment can effectively remove the impurities in the precursor film and promote the formation of lattice network [29,54,55,61,62,63,64,65]. There are usually three mechanisms for the interaction between laser and precursor film: (1) thermal effect of laser; (2) photochemical cleavage of metastable bonds; and (3) photochemical effect. The process of thermal effect of laser usually includes: (1) carrier excitation; (2) carrier–carrier scattering, carrier–phonon scattering, and energy transfer to the lattice due to spontaneous phonon emission; (3) when the carrier and lattice reach equilibrium, the film is heated, as shown in Figure 4 [51,66]. High energy photons can induce the photochemical cleavage of chemical bonds related to metal alkoxy and carbon impurities and promote the subsequent reorganization of metal oxide frames [27,55,63]. These mechanisms are also observed in other light-assisted methods. However, the difference between laser processing and other photo-assisted methods that take a longer time is that laser combines these photochemical effects with laser-induced high temperature heating to provide additional local heat energy. Therefore, the laser treatment can effectively decompose the impurities related to the precursor, remove the metal oxide defects, and reorder the metal oxide structure instantly (<100 ns) at a lower substrate temperature (RT). Laser acting on thin films can not only produce thermal effect through instantaneous high energy radiation, but also produce photoactivation effect by high energy photons [43,53,67,68]. The photoactivation effect is that the residual metal ligands in the precursor films are photolyzed by high energy photons to produce free radicals. The free radicals mediate the reaction to form the M-O-M lattice structure and decompose the chemical impurities into small gas molecules [29], as shown in Figure 5.

Juan et al. used KrF excimer laser with wavelength of 248 nm to treat IZO-TFT and found that excimer laser treatment effectively removed carbon impurities related to the precursor and improved the lattice structure [43]. Chen et al. used a femtosecond laser with a wavelength of 800 nm to treat IZO-TFT. The high-energy photons generated by the laser induced the photo assisted condensation reaction, resulting in the formation of metal oxide bonds by metal hydroxides, and dehydroxylation reaction at the same time to remove residual impurities [62]. Dellis et al. treated In_2_O_3_ thin films with KrF excimer laser and characterized them by X-ray photoelectron spectroscopy (XPS). The degree of conversion of initial precursors to metal oxides was evaluated by the ratio of In-O to In-OH bonds. After laser treatment, the proportion of In-O bond increased significantly, while the proportion of In-OH bond decreased. The results show that laser treatment can effectively promote the transformation of precursors to metal oxides [69]. Fei et al. used femtosecond laser treatment to treat IZO-TFT. They proposed that laser treatment can break In-O and Zn-O bonds and form metal oxygen lattice structures such as In-O-Zn-O or Zn-O-In-O under thermal effect [52].

### 2.2. Active Layer Thin Films Prepared by Nano-Particle Method

**Figure 6:**
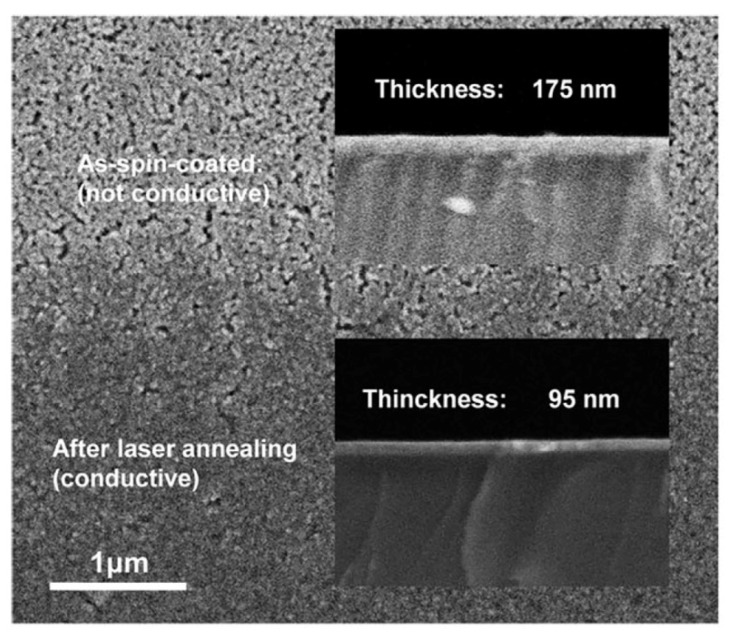
SEM images of ZnO films before and after laser treatment [73].

In addition to sol–gel method, nano-particle method is another common solution method to prepare MOS-TFT active layer. Nanoparticles are prepared by coprecipitation or hydrothermal method and deposited by inkjet printing or rotary coating [44,70]. Laser treatment can provide high temperature up to the melting point of nanoparticles, sinter nanoparticles and form semiconductor films [71,72]. Qion et al. prepared AZO thin films by rotary coating method, and studied the laser sintering process of nano-particles. According to their simulation study, they proposed that in the process of interaction between laser and nanoparticles, the contact zone between nanoparticles is first heated to form hot spots, and then the heat spreads to the interior of the particles and adjacent particles. The hot spots in the contact zone promote the surface melting and merging of the nanoparticles, increase the grain size, change the grain shape and compress the internal gap, and finally form a continuous dense film [71]. Lee et al. prepared ZnO-TFT by nano-particle method and treated with yttrium vanadate (Nd:YVO_4_) picosecond (ps) ultraviolet laser. Their study found that before laser treatment, particles and nano-pores were observed, and the thickness of the film was 175 nm. After laser treatment, the grains are melted, the voids are reduced, and the thickness of the film is reduced to 95 nm, indicating that the film is densified by laser treatment, as shown in Figure 6 [73].

## 3. Application of Laser Treatment in MOS-TFT Performance Control

Combined with the properties of the thin film (film thickness, composition, absorption spectrum, etc.), the physical, optical, electrical and chemical properties of the thin film and MOS-TFT can be adjusted by changing the laser processing parameters (energy density, frequency, treatment atmosphere, etc.). Table 2 summarizes the examples of laser processing of metal oxide semiconductor thin film transistors.

### 3.1. Laser Energy Density

**Figure 7:**
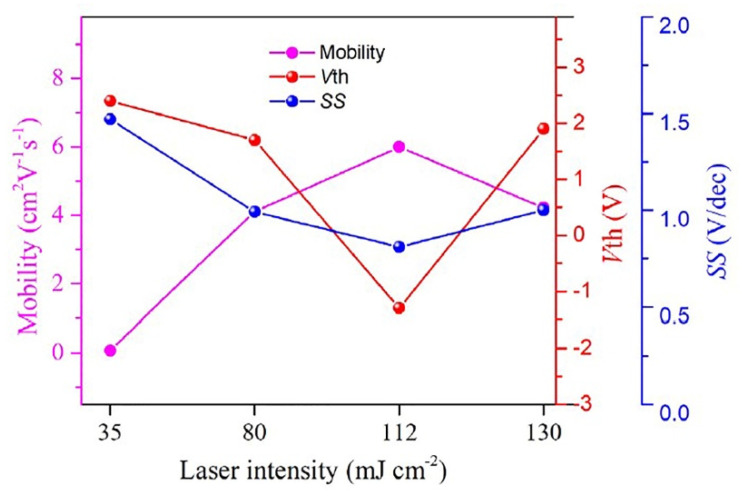
Variation in device performance results of IGZO-TFTs as a function of laser intensity [75].

Laser energy density is an important factor affecting the lattice structure of metal oxide films. When the laser energy density is too low, the film may not be treated effectively, and when the energy density is too high, it may also have an adverse impact on the performance of the device [77]. Chen et al. prepared IGZO-TFT and treated it with femtosecond laser with wavelength of 800 nm and energy density of 20, 35, 80, 112 and 130 mJ/cm^2^. Their research found that the films treated at 20 mJ/cm^2^ energy density will produce serious defects and trap states due to the incomplete transformation of precursors to metal oxide lattice, and the devices do not have TFT characteristics. With the increase of laser energy density, the performance of TFT devices is improved, and the best device performance is obtained at 112 mJ/cm^2^. When the laser energy density increases to 130 mJ/cm^2^, the performance of the device decreases, as shown in Figure 7 [75].

**Figure 8:**
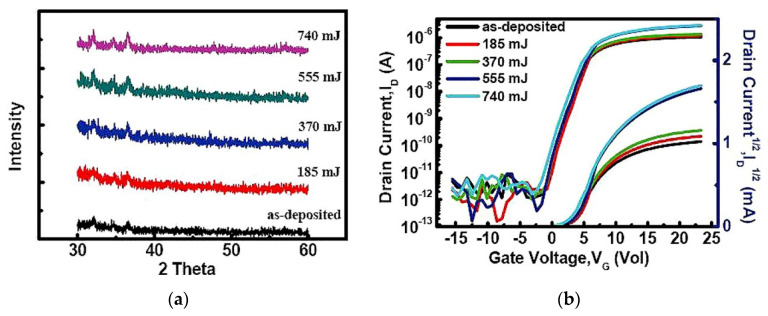
Crystallization degree of ZnO thin films and transmission characteristics of ZnO-TFT devices under different laser energy densities: (**a**) degree of crystallization; (**b**) transmission characteristics [58].

There is usually a certain energy threshold in metal oxide thin films, and the laser energy exceeding the threshold will induce the recrystallization or grain growth of the thin films [68,78,79]. The grain size usually increases with the increase of laser energy density [80,81]. For polycrystalline thin films, the bottleneck of field effect mobility usually occurs at grain boundaries, so reducing the number of grain boundaries and increasing grain size by laser treatment can improve the device performance [60,82,83,84,85]. Nagase et al. studied the effects of laser energy density and film thickness on the properties of ZnO films. Their research found that two kinds of crystal ZnO films were obtained under different laser energy density and different film thickness. Low energy density produces low crystallinity with weak orientation, while high energy density produces high crystallinity with strong orientation, and the threshold of energy density increases with the increase of film thickness [86]. Yang et al. prepared ZnO-TFT and treated it with a Nd:YAG laser with a wavelength of 355 nm. It is found that laser treatment can improve the crystallinity of ZnO materials, and the mobility of TFT devices is increased by more than 2.5 times (0.19 to 0.49 cm^2^/Vs) as shown in Figure 8 [58].

### 3.2. Treatment Atmosphere

**Figure 9:**
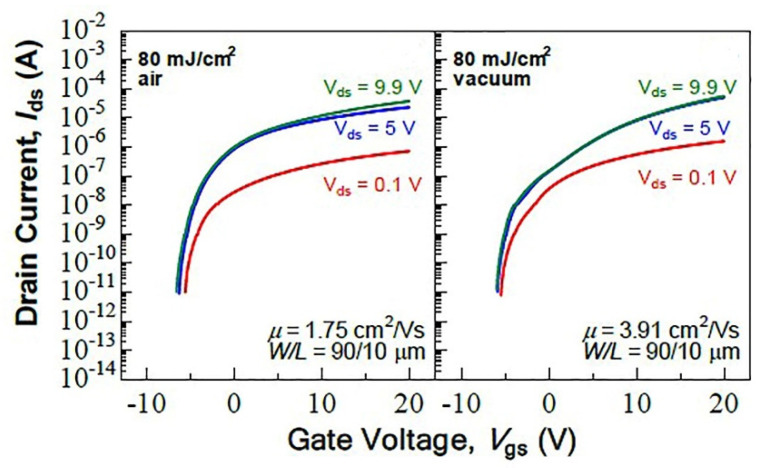
Performance of IZO-TFT devices treated by laser in different atmospheres [43].

Laser treatment in air has the advantages of low cost, simple process and more suitable for large-area manufacturing, but the moisture and oxygen in air will affect the properties of the film [40,87]. During laser treatment, the film surface will be heated to a temperature sufficient to destroy the M-O bond and form an oxygen vacancy [88,89,90]. If treated in an air atmosphere, the oxygen in the air will oxidize the metal elements in the film again to form an M-O bond. The destruction rate of M-O bond and the oxidation rate of metal elements together determine the concentration of oxygen vacancies in the thin films. Usually, the formation of oxygen vacancies is often accompanied by the generation of electrons, which increases the carrier concentration of metal oxide films [68,91]. The increase of oxygen vacancy concentration causes high carrier concentration to form an electron transport path near the conduction band, thus increasing the mobility of TFT devices and reducing the threshold voltage [76,92,93,94,95]. However, too high oxygen vacancy concentration may lead to high leakage current of TFT devices due to high carrier concentration, which reduces the device performance [52]. Therefore, by adjusting the gas atmosphere during laser treatment, the concentration of oxygen vacancies in the thin films can be effectively controlled and the electrical properties of MOS-TFT devices can be improved. Lee et al. studied the effects of ambient atmosphere and argon atmosphere on the laser-treated ZnO thin films. Their study found that the oxygen vacancy content of the films treated in argon atmosphere was significantly higher than that in the ambient atmosphere [73]. Juan et al. treated IZO-TFT with KrF excimer laser in air atmosphere and vacuum. Their study found that laser treatment in vacuum can inhibit the absorption of extra water and excess oxygen from the atmosphere, and the device mobility is higher than that of laser treatment in air, as shown in Figure 9 [43].

### 3.3. Laser Wavelength

**Figure 10:**
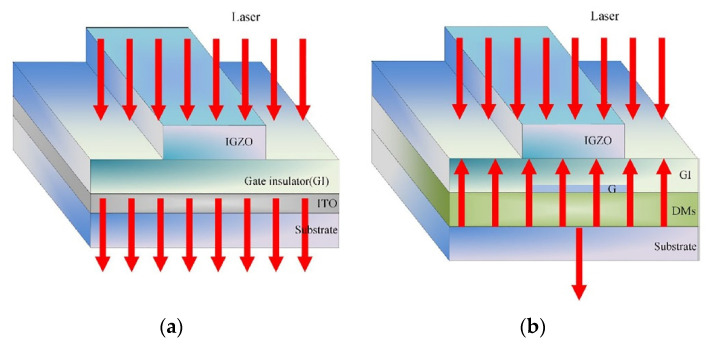
Protective effect of DMs on flexible substrate: (**a**) without DMs; (**b**) with DMs [75].

Due to laser processing is a top-down process, according to Beer-Lambert law, the radiation intensity of laser attenuates in the film [96,97]. The shorter the laser wavelength is, the shallower the penetration depth is. Therefore, in the process of laser treatment, temperature gradients are easy to exist in the film, resulting in differences in the properties of regions with different depths of the film. This effect is particularly significant in the films treated by ultraviolet wavelength lasers [55,98,99]. Kwon et al. prepared ZTO thin films by sol–gel method and treated them with KrF excimer laser. They performed high-resolution chemical and microstructure analysis of the films. Their study found that during the UV laser treatment, the top temperature of the ZTO film is much higher than that in the deep region. This temperature gradient makes the Zn element enriched in the surface region and the Sn element enriched in the bottom region [55]. Sandu et al. found that due to the different penetration thickness of SnO_2_ thin films by 193 nm and 248 nm laser (66 nm and 148 nm, respectively), the crystallization effect is different, and the crystal gradient of the thin film irradiated by 193 nm laser is more obvious [80,81]. In contrast to the UV laser, the infrared laser has a long wavelength and a large penetration depth in the film, which can heat the film more evenly. However, due to its deep penetration depth, when applied to flexible MOS-TFT, the flexible substrate may be damaged by a large number of high-energy photons, which will affect the performance of MOS-TFT [9,51,75,100]. Chen et al. fabricated MOS-TFT devices and embedded dielectric mirrors (DMs) in them. Their research shows that the DMs can effectively prevent the penetration of high energy photons into the PEN substrate, thus avoiding the damage to the substrate and significantly improving the performance of the device, as shown in Figure 10 [75].

## 4. Conclusions and Prospects

The excellent compatibility between laser processing technology and solution method has been widely recognized. In the sol–gel method, laser treatment can effectively remove impurities in the precursor films and promote the formation of lattice networks. In the nano-particle method, the nano-particles are sintered effectively by laser treatment to form a continuous and dense film. The performance parameters of thin film and MOS-TFT can be effectively improved by adjusting various parameters in the process of laser processing. Although laser processing technology has made remarkable achievements in the application of active layer thin films and MOS-TFT devices prepared by solution method, there are still some deficiencies in the production of flexible, large-size, low-cost MOS-TFT and further improving the performance of MOS-TFT devices, which need to be further studied and improved. These include (1) less research on flexible devices; (2) less research combined with other low temperature treatment processes. (3) present research only focused on the effect of different laser energy density on thin films; there are few studies on other parameters of laser treatment, such as pulse number, frequency and so on; (4) the research on the mechanism and physical model of the interaction between laser and thin film is not deep enough. In the future, by using different flexible substrates, adjusting the process parameters of laser processing, and combining laser processing with other low temperature treatment processes, study of the application of laser processing technology in active layer thin films and MOS-TFT devices prepared by solution method, so as to promote the development of flexible large size display technology should continue.

## Figures and Tables

**Table 1:** Low temperature treatment process parameters.

Treatment Method	Treatment Temperature	Treatment Time	Refs.
Microwave treatment	>180 °C	>30 min	[22,23,24]
Plasma treatment	>300 °C	>20 min	[23,25]
Ozone ultraviolet treatment	>120 °C	>5 min	[26,27,28,29,30]
High pressure treatment	>220 °C	>1 h	[22,23,31,32]
Water based/hydrolysis	>230 °C	>2 h	[28,33]
Low temperature steam treatment	>220 °C	>1 h	[34]
Laser treatment	>95 °C	<5 min	[38,39,40,41,42,43]

**Table 2:** Examples of laser treatment of MOS-TFT.

Channel Material	Solution Type	Laser Wavelength (nm)	μ (cm^2^ V^−1^ s^−1^)	SS (V dec^−1^)	On/Off Ratio	Ref.
IGZO	Sol-gel	355	7.65			[74]
IGZO	Sol-gel	800	4.24	0.91	7.2 × 10^5^	[75]
ZnO	NPs	355	0.5		1.7 × 10^6^	[53]
IGZO	NPs	355	7.65		2.71 × 10^6^	[53]
IGZO	Sol-gel	1064	1.5		1.29 × 10^6^	[76]
In_2_O_3_	Sol-gel	700	10.03 ± 0.64	1.44 ± 0.37	3.4 × 10^5^	[63]
In_2_O_3_	Sol-gel	248	13		10^6^	[69]
IZO	Sol-gel	800	3.75	1.21	1.77 × 10^5^	[52]
IZO	Sol-gel	248	0.58			[47]
ZnO	NPs	355	3.01	1.8	10^5^	[73]
